# New protein-DNA complexes in archaea: a small monomeric protein induces a sharp V-turn DNA structure

**DOI:** 10.1038/s41598-019-50211-2

**Published:** 2019-10-03

**Authors:** Karine Loth, Justine Largillière, Franck Coste, Françoise Culard, Céline Landon, Bertrand Castaing, Agnès F. Delmas, Françoise Paquet

**Affiliations:** 1 Centre de Biophysique Moléculaire, Centre National de la Recherche Scientifique UPR 4301, rue Charles Sadron, F-45071 Orléans, Cedex 2 France; 2 UFR Collegium Sciences et Techniques, Université d’Orléans, rue de Chartres, 45100 Orléans, France

**Keywords:** DNA, DNA-binding proteins, Solution-state NMR

## Abstract

MC1, a monomeric nucleoid-associated protein (NAP), is structurally unrelated to other DNA-binding proteins. The protein participates in the genome organization of several *Euryarchaea* species through an atypical compaction mechanism. It is also involved in DNA transcription and cellular division through unknown mechanisms. We determined the 3D solution structure of a new DNA-protein complex formed by MC1 and a strongly distorted 15 base pairs DNA. While the protein just needs to adapt its conformation slightly, the DNA undergoes a dramatic curvature (the first two bend angles of 55° and 70°, respectively) and an impressive torsional stress (dihedral angle of 106°) due to several kinks upon binding of MC1 to its concave side. Thus, it adopts a V-turn structure. For longer DNAs, MC1 stabilizes multiple V-turn conformations in a flexible and dynamic manner. The existence of such V-turn conformations of the MC1-DNA complexes leads us to propose two binding modes of the protein, as a bender (primary binding mode) and as a wrapper (secondary binding mode). Moreover, it opens up new opportunities for studying and understanding the repair, replication and transcription molecular machineries of Archaea.

## Introduction

In the three domains of life, DNA-binding proteins are involved in genome organization, packaging it into eukaryotic organelles or into the cell (in bacteria or archaea), while efficiently accommodating DNA-based processes such as transcription, replication and repair. Each organism has a characteristic set of DNA-binding proteins that has unique regulatory features. Advances in this field have undoubtedly benefited from high-resolution structures of DNA-protein complexes, as well as analyses of the global and local effects of these proteins on chromatin structure.

DNA-binding proteins are classified in three categories: benders, bridgers and wrappers^1^. Focusing on archaea, Alba and histone proteins are the most widely represented^2–4^. They belong to the groups of bridgers and wrappers, respectively. Alba is present in all archaea, except in two classes of *Euryarchaea* (*Methanomicrobia* and *Haloarchaea*). In archaea lacking Alba, the Methanogen Chromosomal protein 1 (MC1) is present. Alba shows a bimodal DNA-binding behavior by bridging or stiffening the DNA^5^. The Alba family of proteins exhibits functional diversities: genome packaging and organization, transcriptional and translational regulation (through acetylation/deacetylation mechanisms), RNA metabolism, development and differentiation processes^6^. Histone proteins are present in all *Euryarchaea* (except *Thermoplasmatales*) and in some *Crenarchaea*. The homo- and heterodimers of HMfA and HMfB proteins, from the hyperthermophile *Methanothermus fervidus*, are the benchmark of the wrapper histone proteins^7^. They oligomerize in the presence of DNA, further forming tetramers and even hexamers. Besides, in order to compact DNA, histone proteins modulate transcriptional regulation by interplaying with transcription factors^8^.

Several other archaeal nucleoid-associated proteins (NAPs) belonging to the benders are less widely distributed among the archean phyla. In *Crenarchaea* lacking histone proteins, Cren7 is always present whereas Sul7 (also known as Sso7d or Sac7d) is restricted to *Sulfolobus*^8,9^. They non-specifically bind to DNA by inserting hydrophobic residues into the minor groove, bending the DNA by up to 60° ^10–12^. HTa and HUTvo belonging to the HU structural superfamily are found in *Thermoplasma* species, which are the only *Euryarchaea* lacking histone proteins^13,14^. The dimeric HU-family proteins present a compact core of α-helices with two emerging flexible β-ribbon arms^14^. They form low-affinity HU-DNA complexes without sequence specificity and high-affinity complexes with flexible or bent DNA, such as single-strand nicks and gaps, and bulged and branched DNA structures^15–17^.

The MC1 protein studied here belongs to *Methanosarcina thermophila*. In laboratory growth conditions, MC1 is the most abundant architectural protein in *Methanosarcina thermophila* CHTI55^18^ and was primarily classified as histone-like protein^19–21^. Depending on the growth conditions, MC1 could compact and organize DNA in a number of different ways. It was also proposed to be involved in DNA transcription (*in vitro* essays)^22^ and cellular division (proteomics by LCMS)^23,24^, through as yet unknown mechanisms. This small basic monomeric protein of 93 residues (UniProt P12770) is structurally unrelated to other DNA-binding proteins^25,26^. Its affinity, determined by gel retardation essays under direct competitive conditions, for any double-stranded DNA is high (K_D_ ~10^−7^ M) without strong sequence specificity but with a conformational preference for bent DNA, such as negatively supercoiled DNA minicircles (K_D_ <10^−8^ M)^20^ and four-way junctions (K_D_ ~2.5 10^−8^ M)^27^. Binding of two MC1 molecules per DNA minicircle induces two diametrically opposed kinks visible by electron microscopy^21^ and binding of MC1 to linear 176 base pairs (bp) DNA duplexes leads to a bend angle estimated to be 116° ^19^. When DNA is bound to MC1, it is protected against thermal denaturation^18^ and radiolysis^28^.

Recently, combining NMR and biochemical data through data-driven docking, we proposed an atypical compaction mechanism of DNA by MC1^29^. We describe here the solution structure of an MC1-DNA^15bp^ complex, uncovering the first structure of a monomeric protein bound to the concave side of a strongly bent and distorted DNA (PDB code 2NBJ). This new data allowed us to establish that MC1, as a monomer, is able to induce a V-turn on DNAs. The existence of such a conformation opens new insights into MC1-related and MC1-based biological processes, including transcription, replication and repair.

## Results

In this work, a new type of a protein-DNA complex structure, formed by MC1 and a DNA^15bp^ was determined using NMR spectroscopy data. To avoid overlaps in NMR resonance peaks, we chose the [AAAAACACACACCCA] DNA^15bp^ sequence from the consensus [AAAAACACAC(A/C)CCC(C/A)] sequences revealed by a SELEX (systematic evolution of ligands by exponential experiment) procedure^30^.

We observed six NOEs between the protons of the side chain of Pro72 with the sugar protons of T_16_, G_17_ and G_18_ located in the minor groove. Twelve other NOEs were unambiguously identified to the side chain protons of Trp74 (HB1, HB2, HD1, HE3 and HZ2) in contact with H2 and sugar protons of A_15_ (H1′) and T_16_ (H2′, H3′, H5′) and amino protons H21/22 of G_17_, all located in the minor groove. Moreover, we observed eight NOEs between the side chain protons of Ile89 (HB, HD1#) and the sugar protons of A_5_ (H5′) and T_30_ (H1′, H4′, H5′and H5″) located in the minor groove. The amino side chain of Gln23 gave observable NOEs with sugar protons of T_22_ and amino protons of C6 located in the major groove. The use of NOE restraints is generally not sufficient to determine global structural features such as bending of nucleic acids. RDC constraints, known to improve the precision and accuracy of both the local and global structures of the double helix^31,32^, were measured on both the DNA (the labelled strand) and protein. Their addition in the calculation was an absolute necessity to resolve the 3D structure of the complex.

**Figure 1:**
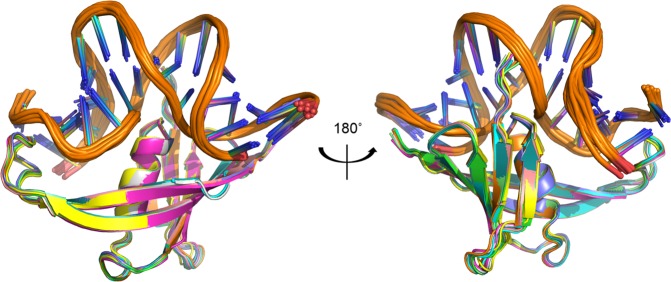
Overlay of the twelve-deposited structures of the MC1-DNA^15bp^ in cartoon representation. The rmsd for all heavy atoms of the complex is equal to 0.7 Å.

The type and number of restraints, and the structural statistics for the twelve structures deposited to the PDB are reported in Table 1. Superimposition of these structures clearly shows that they are well defined (Fig. 1).

**Table 1:** Experimental restraints and structure statistics for MC1-DNA^15bp^ complex.

Number of experimental restraints				
*Distance restraints from NOEs*		*1209*		
MC1			735	
DNA^15bp^			424	
Intermolecular			50	
*H-bonds*		*142*		
MC1 (secondary structures)			70	
DNA (base-pairing)			72	
Intermolecular			0	
*Residual dipolar coupling (RDC)*		*113*		
MC1			82	
DNA^15bp^			31	
Average violations per structure*				
*Average distance restraint violations* > 0.5 Å				
MC1			10.2 (1.4%)	
DNA^15bp^			0.3 (0.07%)	
Intermolecular			0.6 (1.2%)	
*H-bonds* > 0.5 Å				
MC1 (secondary structures)			0	
DNA^15bp^ (base-pairing)			0	
RDC constraints rmsd (Hz)				
MC1			0.61	
DNA^15bp^			0.86	
Ramachandran analysis of MC1				
Residues in favored regions (%)			68.4	
Residues in additional allowed regions (%)			24.4	
Residues in generously allowed regions (%)			4.1	
Residues in disallowed regions (%)			3.1	
Average pairwise rmsd (Å)	Backbone			All heavy atoms
MC1	0.45 ± 0.10			0.67 ± 0.10
DNA^15bp^	0.76 ± 0.15			0.72 ± 0.13

*No distance restraint violation observed at 0.7 Å and only an average of eleven non-recurring violations at 0.5 Å (ten for the protein and one intermolecular).

### MC1 slightly adapts its conformation upon DNA binding

**Figure 2:**
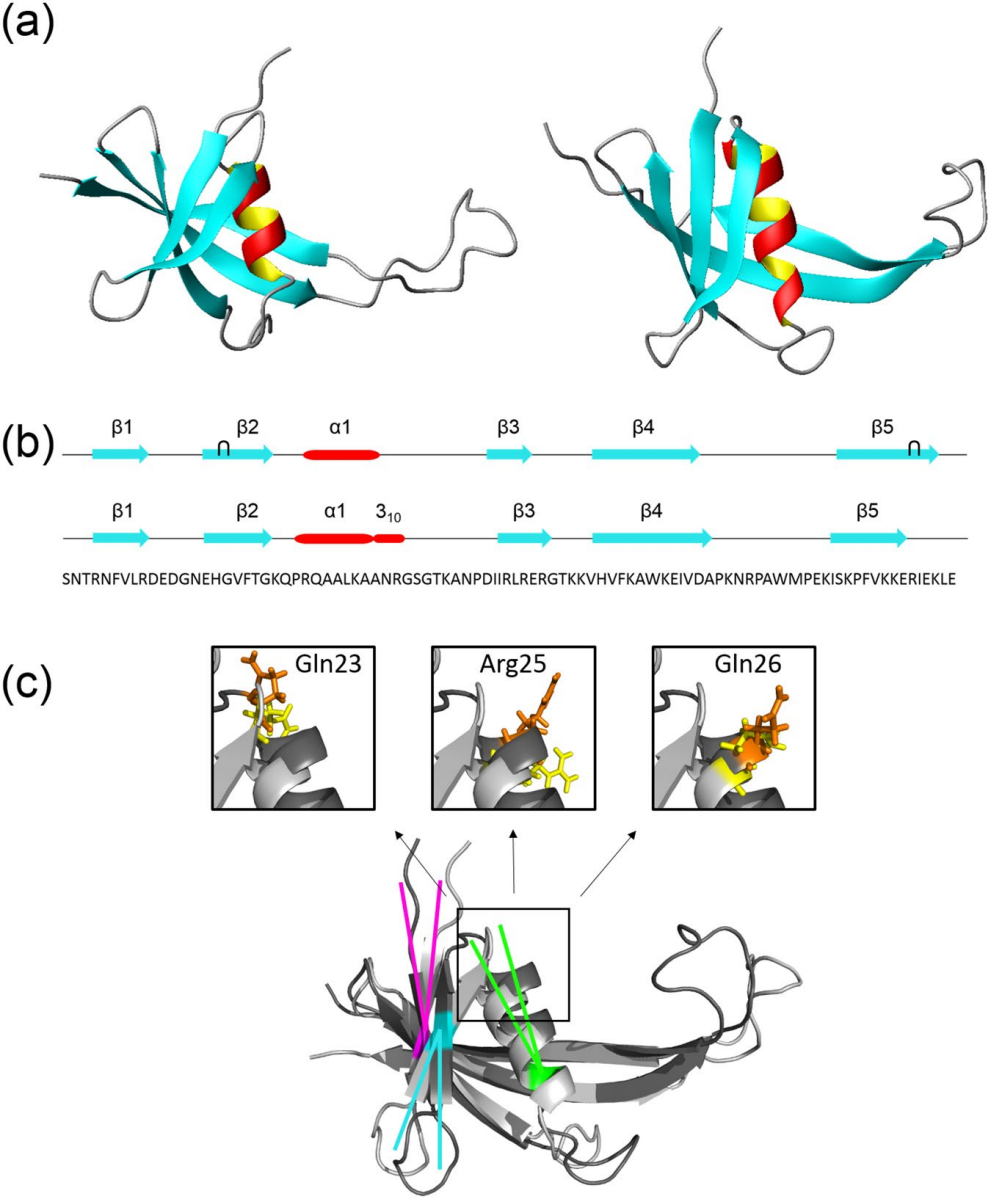
MC1 slightly adapts its conformation upon binding to the DNA^15bp^. (**a**) 3D solution structures of MC1 in its free (left) and bound (right) forms in cartoon representation. The β-strands and the α-helix are colorized in blue and red, respectively. (**b**) Primary and secondary structures of MC1 free (top) and bound (bottom): Blue arrows and red cylinder are for β - and α -structures, respectively, and black wickets for bulges. (**c**) Overlay of the cartoon representations of the free (dark grey) and bound (light grey) conformations of the MC1 protein. In order to highlight the conformational changes of MC1 upon binding, three couples of axes are represented: rotation of the α-helix by 10° around Ala32 in green, of the β1-strand by 10° around Val7 in magenta and of the β2-strand by 30° around Val18 in cyan. Close-up pictures of the side chains of Gln23, Arg25 and Gln26, in yellow (free MC1) and orange (bound MC1), show that their orientation have changed upon binding in order to be optimally positioned for an interaction with DNA. Alignment of the two MC1 structures, in their free and bound forms, give a rmsd of 4 Å (all residues) and a rmsd of 2.3 Å (without the arm).

Small conformational changes of the 3D structure of MC1 occur upon DNA binding (Fig. 2a). In its free form, secondary structure elements of MC1 consist of an α-helix (Arg25-Ala32) and two β-sheets^26^. The first sheet is composed of two antiparallel β-strands, β1 (Arg4-Arg9) and β2 (Glu15-Gly21), while the second contains three antiparallel β-strands β3 (Asp43-Arg48), β4 (Val55-Val65), and β5 (Ile79-Glu90). Each β-sheet contains one antiparallel β-bulge composed of Leu8, His16, Gly17 and Val57, Glu87, Arg88, respectively (Fig. 2b). Upon DNA binding, the helix of MC1 appears longer and is, indeed, composed of an α-helix (Pro24-Ala31) followed by a small 3_10_ helix (Ala32-Arg34). The helix rotates by 10° around Ala32, the β1-strand by 10° around Val7 and the β2-strand by 30° around Val18, leading the side chain of Gln23, Arg25 and Gln26 to be optimally positioned for an interaction with DNA (Fig. 2c). Due to the displacement of both β1 and β2 strands, a unique five β-stranded antiparallel β-sheet is formed, and the first β-bulge (Leu8, His16, and Gly17) disappears. The second β-bulge (Val57, Glu87 and Arg88) also disappears, due to interactions of Lys86, Arg88 and Ile89 (β5-strand) in the DNA minor groove. Furthermore, MC1 adopts an extended and less mobile conformation of the arm (Ala67-Glu77) due to its binding to DNA^26^.

### MC1-DNA^15bp^ interactions

**Figure 3:**
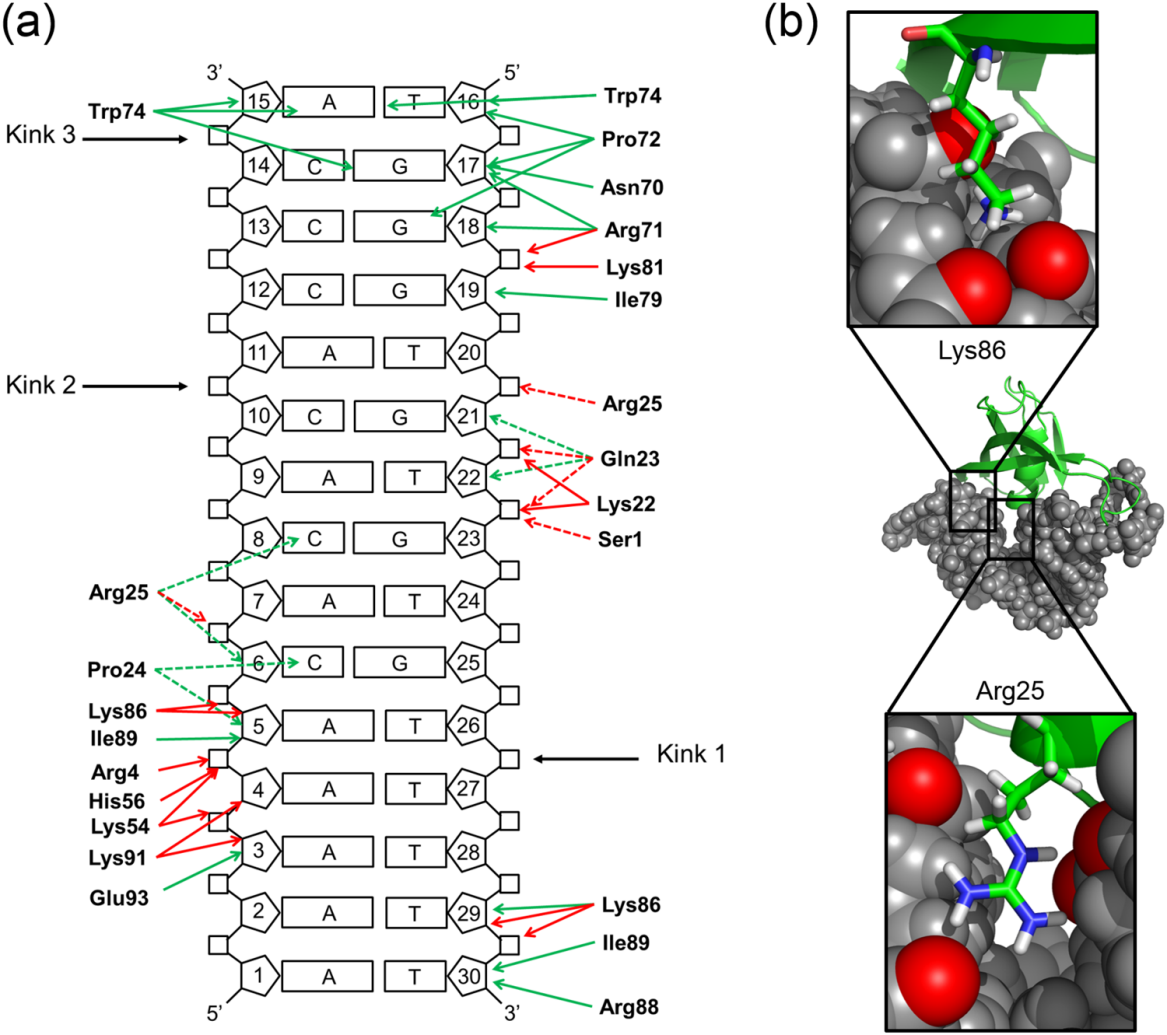
(**a**) Interactions between MC1 and DNA^15bp^. Green and red arrows represent observed NOEs and H-bonds formed in the complex, respectively. Dashed arrows are for interactions in the major groove and standard arrows for interactions in the minor groove. Kinks are represented by black arrows. (**b**) Neutralization of the phosphates by side chains of Lys86 and Arg25 is essential to the compression of the minor and major grooves, respectively.

MC1 binds the DNA through two main areas of contact into the minor groove of the DNA (Fig. 3a). The first one, in the A-tract, is dominated by the formation of hydrogen bonds between the side chains of Lys54, His56, Lys86 and Lys91, and DNA phosphate groups (Fig. 3b). Note that the side chain of Arg4 interacts in the major groove and Van der Waals’ interactions occurring between the non-polar side chain of Ile89 and sugar groups of A_5_ and T_30_ strengthen these interactions. The second is dominated by hydrophobic interactions through the insertion of Pro72 and the intercalation of Trp74 between the C_14_pA_15_ step. To reinforce these interactions, Arg71 and Lys81 make electrostatic contacts with the sugar-phosphate backbone of G_18_pG_19_. Between these two areas of contact, the DNA is composed of a more flexible sequence (CpA)_3_ in which the major groove is compressed. The presence of the positive side chain of Arg25 is essential to neutralize the repulsive negative charges of the phosphates belonging to the closely spaced C_6_pA_7_ and T_20_pG_21_ steps in the major groove (Fig. 3b). The side chains of Lys22, Gln23 and Gln26 participate in the stabilization of the complex by forming hydrogen bonds with the sugar-phosphate backbone of G_21_pT_22_pG_23_ steps, Gln26 in the minor groove, Gln23 in the major groove and Lys22 either in the minor or the major groove depending on the structures. Residues essential to DNA-binding and -bending were highlighted and confirmed by site-directed mutagenesis^29^. MC1, as many NAPs, interacts with DNA using a combination of the two main and often interrelated readout mechanisms: recognition of the DNA shape (e.g., narrow A-tract minor groove, wide CpCpC major groove, kinking, bending) and recognition of bases (e.g., hydrogen bonds and hydrophobic interactions into the major and minor grooves)^33^.

### MC1 imposes a severe bend and a torsion on DNA^15bp^ leading to a V-turn DNA structure

**Figure 4:**
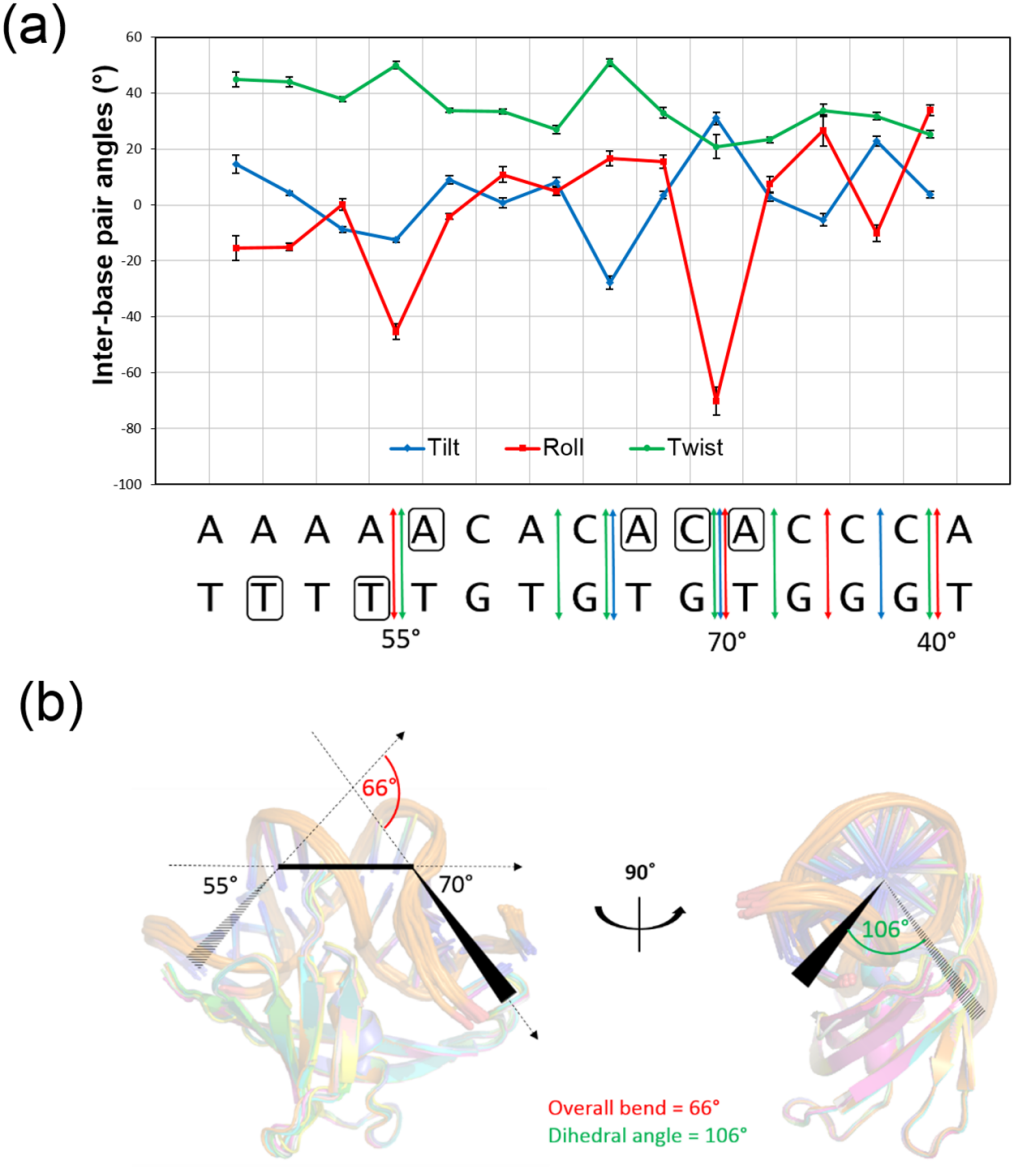
(**a**) Top: Tilt (blue), roll (red) and twist (green) angles profiles in the MC1-DNA^15bp^ structure. Bottom: DNA^15bp^ sequence, colored arrows summarized the base pairs parameters differing from standard values, boxed letters indicate sugars in north pucker conformations and values of the bend angles are given. (**b**) Superimposition of the 12 deposited structures of the MC1-DNA^15bp^ in cartoon representation. The two kinks are represented as well as the torsional angle.

The MC1-DNA^15bp^ complex reveals large conformational perturbations in the DNA structure, which exhibits a dramatic bending, unstacked base pairs and anomalous groove widths. The DNA conformation analysis with Curves+^34,35^ and 3DNA^36^ programs cannot correctly define the base-pair parameters and the geometry of the grooves because of the very unusual conformation of the DNA, which cannot be easily classified as a B- or an A-DNA conformation. However, the roll angle profile shows two sharp peaks, which reflect distortions of base stacking owing to acute DNA bending by two kinks: the first of (55 ± 2)° between A_4_pA_5_/T_26_pT_27_ (<tilt> = −13°, <roll> = −45°, <twist> = 50°), and the second of (70 ± 3)° between C_10_pA_11_/T_20_pG_21_ (<tilt> = 31°, <roll> = −71°, <twist> = 20°). Negative roll results in bending of the oligonucleotide toward the minor groove, which becomes very narrow at the kinks (Fig. 4a). These two bend angles, resulting principally from large tilt angles at the site of kinking, are not coplanar and create a dihedral angle of (106 ± 3)° (Fig. 4b). Between the two kinks, another striking feature is the C_8_pA_9_/T_22_pG_23_ junction (<tilt> = −28°, <roll> = 17°, <twist> = 50°, A_9_ in north pucker) which leads a smooth bend towards the major groove. From A_7_ to A_9,_ the minor groove is particularly wide and the major groove abnormally narrow.

In agreement with the ability of MC1 to restrain negative supercoils, our structure shows that MC1 introduces both unwinding and negative writhe^37^. Positive base pair tilt values and/or north pucker of the sugars are characteristic of an A-DNA-like structure. A_1_pA_2_/T_29_pT_30_, A_5_pC_6_/G_25_pT_26_, C_10_pA_11_/T_20_pG_21_ and C_13_pC_14_/ G_18_pG_19_ exhibit such tilt values and the sugars of A_5_, C_10_, A_11_, and T_27_ adopt a north pucker (C3′-endo, C2′-exo and C4′-exo conformations). It is known that the B to A transformation leads to a rotation of −3.3° for every transformed base-pair, resulting in an overall untwisting of DNA^38^. Moreover, the average twist in the MC1-DNA^15bp^ is 35°/bp compared with ~36°/bp or ~32.7°/bp for a relaxed B-form DNA and a relaxed A-form DNA, respectively. Although the average twist is close to that of a relaxed B-form, unwinding is locally observed around A_7_pC_8_/G_23_pT_24_, C_10_pA_11_/T_22_pG_23_ and A_11_pC_12_/G_19_pT_20_ steps with twist angle values lower than 30°. As the two kink angles are not coplanar (*vide supra*), the orientation of the resulting torsional angle is consistent with a negative supercoiling.

**Figure 5:**
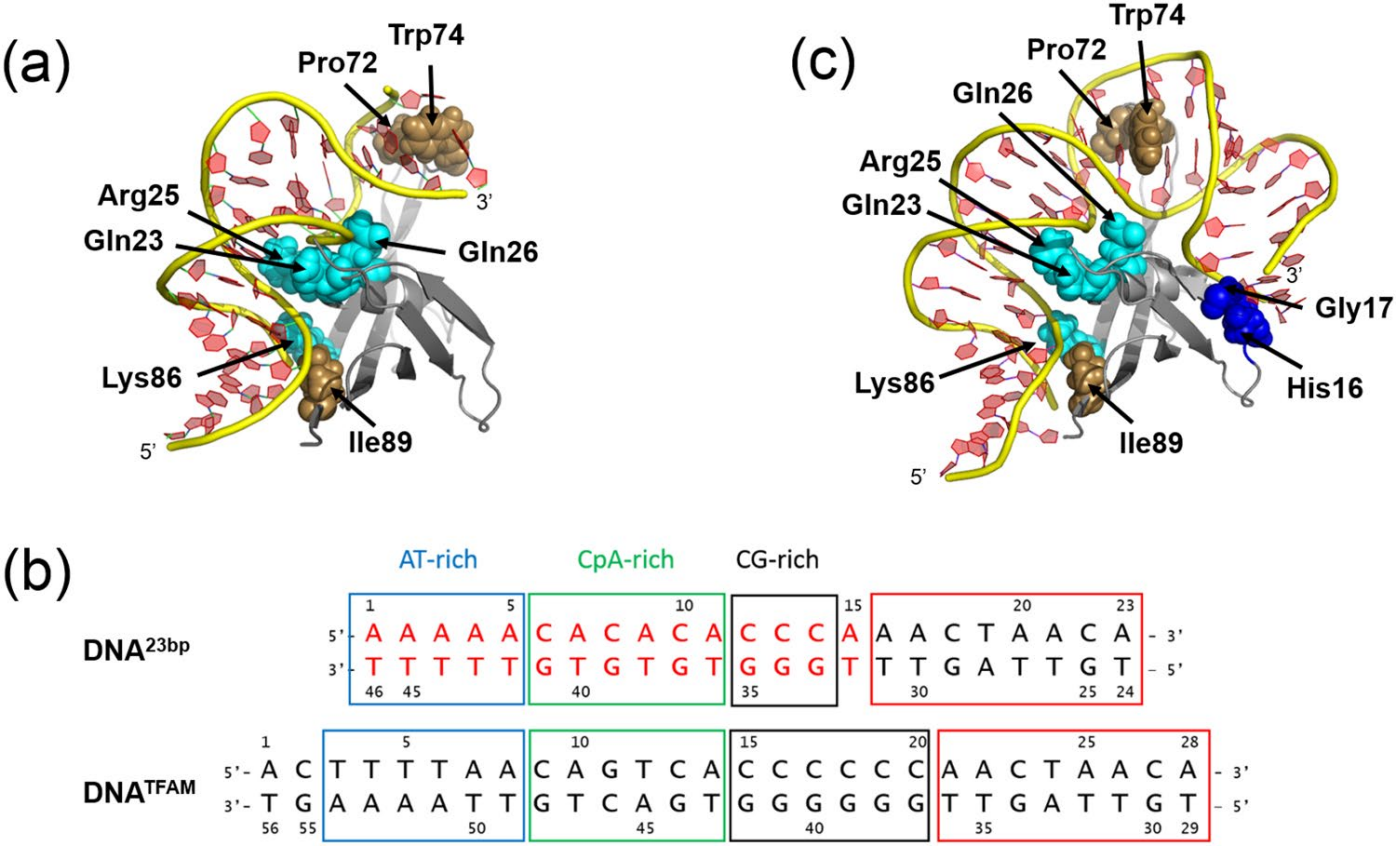
(**a**) Cartoon representation of the MC1-DNA^15bp^ complex. Side chains of key residues are represented as spheres (blue for hydrophilic and brown for hydrophobic residues, respectively). The side chains of Pro72, Trp74, Lys86 and Ile89 contact the minor groove of the DNA, whereas the side chains of Gln23, Arg25 and Gln26 contact the major groove of the DNA. (**b**) Sequences of the chimeric DNA^23bp^ and the DNA^TFAM^. The first 15 base pairs of the chimeric DNA^23bp^ are identical to those of the DNA^15bp^ (written in red). Both sequences have three domains, an AT-rich (blue boxed), a CpA-rich (green boxed) and a CG-rich (black boxed) domains. The eight base pairs chosen from DNA^TFAM^ to lengthen our DNA^15bp^ into DNA^23bp^ are red boxed. (**c**) Cartoon representation of the model of the MC1-DNA^23bp^ complex. New contacts in the DNA minor groove are predicted for His16 and Gly17 side-chains (blue spheres).

A third kink evaluated around 40° occurs between the two terminal base pairs C_14_pA_15_/T_16_pG_17_ upon the insertion of Pro72 and the intercalation of Trp74 (Fig. 5a). As A_15_ and T_16_ are unpaired, the intercalation of Trp74 implies a great perturbation in the stacking with the previous base pair preventing the correct determination of the associated base pairs parameters (tilt, roll and twist). To further extend our exploration of the DNA conformations upon MC1 binding, we decided to study a complex with a longer DNA.

### A third contact observed with a longer DNA: dynamic nature of the V-turn structures

We took inspiration in the U-turn formed by the DNA^TFAM^ promoter upon interaction with TFAM, the major mitochondrial NAP comprising a tandem of HMG-box domains^39^. Indeed, the sequence of DNA^TFAM^ has three domains (AT-rich, CpA-rich and CG-rich domains) similar to DNA^15bp^ and a supplementary domain of 8 bp which was chosen to lengthen our DNA^15bp^ to DNA^23bp^ (Fig. 5b). A structural model of the MC1-DNA^23bp^ complex was built starting from the MC1-DNA^15bp^ complex structure and new interactions with His16, and Gly17 were observed in the minor groove (Fig. 5c). This supplementary contact point, due to the bend and the torsion of the DNA, stabilizes another V-turn conformation of the DNA.

**Figure 6:**
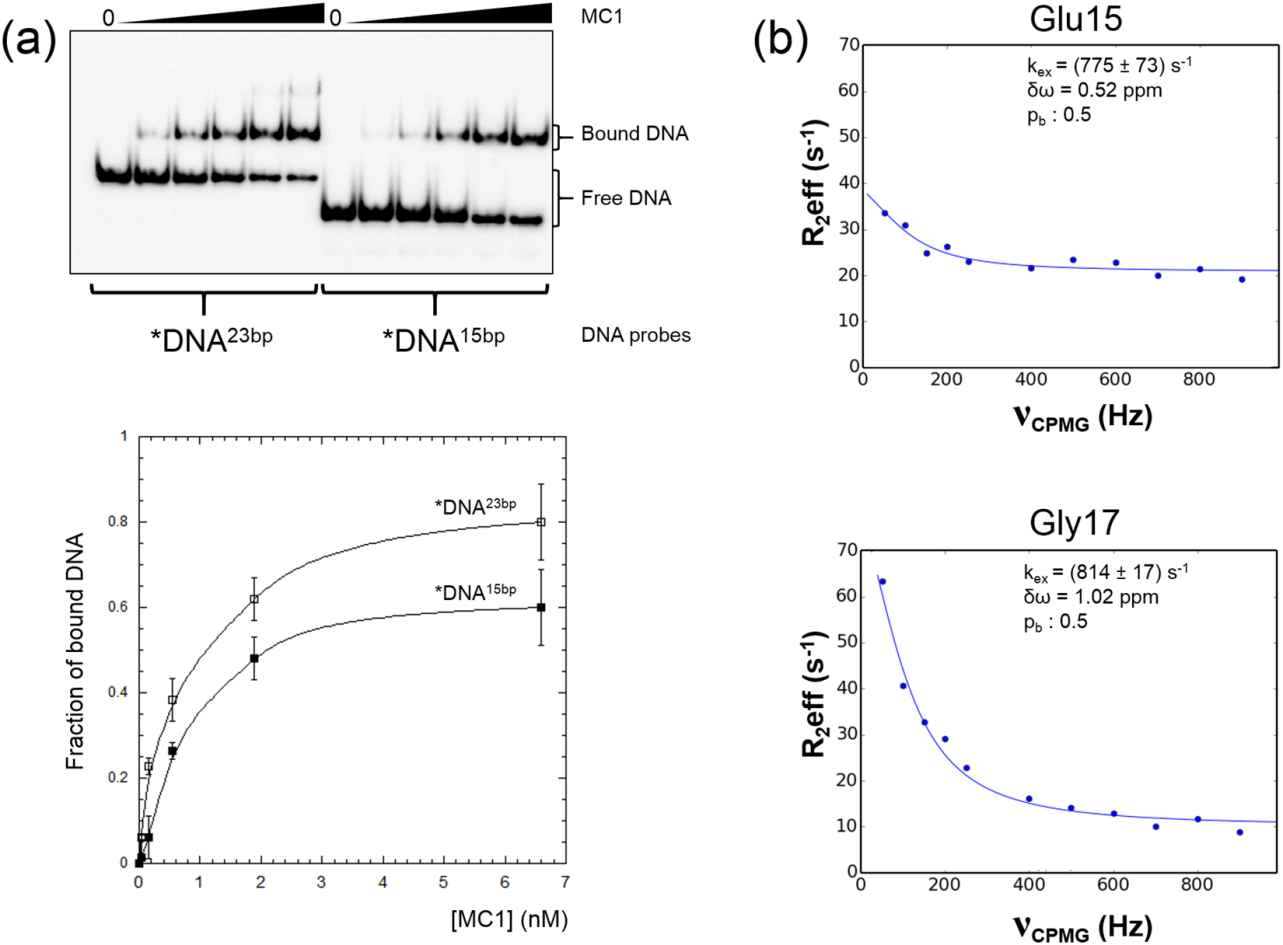
Binding of MC1 protein to two different DNA probes of 17 bp (*DNA^15bp^) and 25 bp (*DNA^23bp^). (**a**) Top: Electrophoresis gel of EMSA reaction mixtures (10 µl) prepared at 4 °C by mixing DNA (0.05 nM) and increasing MC1 protein concentrations from 0.04 nM to 6.6 nM. Bottom: Binding curves obtained from quantification using ImageQuant software of the EMSA experiments. The fraction of bound DNA is plotted as a function of protein concentration [MC1]. (**b**) Relaxation dispersion curves, fitted using the NESSY software, for Glu15 and Gly17 of the MC1 protein in complex with the DNA^23bp^. Data were fitted to a two-site, fast exchange model where *p*_*a*_ and *p*_*b*_ are the populations of the two state models (*p*_*a*_ + *p*_*b*_ = 1), *k*_*ex*_ is the exchange constant and *δω* is the chemical shift difference between states.

We validated the use of the chimeric DNA^23bp^ using EMSA (electrophoretic mobility shift assay) experiments (Fig. 6a). A 2.3-fold increase in affinity to MC1 was observed when the DNA was 8 bp longer (K_Dapp_ = (1.2 ± 0.5) nM for MC1-*DNA^23bp^ and K_Dapp_ = (3.1 ± 0.6) nM for MC1-*DNA^15bp^). This strongly suggested that additional protein residues are involved in the interaction with DNA^23bp^.

A ^15^N-HSQC spectrum of the MC1-DNA^23bp^ complex revealed that this longer DNA affects both the local environment and the dynamics of the protein. Chemical shift perturbations (CSPs) between the free protein and the protein bound to DNA^23bp^ were calculated for all of the observable residues. We observed CSPs for all of the residues already highlighted in the MC1-DNA^15bp^ complex^26^ but most importantly, new CSPs were observed for Val7, Arg9, Glu15, His16 and Gly17. Moreover, some peaks were broader than expected, leading us to hypothesize that the protein is in an intermediate exchange between at least two conformations at the NMR timescale^40^. In particular, the Gln26, Lys30, Lys54, Trp74, Met75, Lys81, Phe83 and Lys86 residues exhibited such a broadened linewidth that they were undetectable. To probe the intermediate chemical exchange regime for the bound MC1, (^1^H-^15^N) CPMG relaxation dispersion experiments were performed. Unfortunately, the resonances of some key residues, especially His16, were overlapped, preventing us from obtaining reliable measurements. However, a slow intermediate chemical exchange was observed for Glu15 and Gly17 with k_ex_ of (775 ± 73) s^−1^ and (814 ± 17) s^−1^, respectively (Fig. 6b). Both residues were in exchange between two equally populated conformations, one close to the MC1-DNA^15bp^ complex structure (longer DNA but only two contact areas) and one corresponding to a different V-turn in which MC1 is bound to DNA^23bp^ with a supplementary contact point.

## Discussion

The 3D structure of the MC1-DNA^15bp^ complex uncovers a very strong bend in the DNA^15bp^ featuring an unprecedented torsional angle of 106°, and a V-turn DNA conformation. It is the first time that atomic data enable to corroborate data from electron micrographs showing the sharp bend angle of 116° in MC1-DNA^176*bp*^ complexes^19^.

The combination of results from our previous^26^ and present studies agree with a three-step mechanism of complexation. Firstly, the dynamic nature of the free DNA^15bp^ structure enables MC1 to transiently select narrow minor groove segments (A-tract), which can be further stabilized through Coulombic interactions with the electropositive binding surface of the protein (Arg4, Lys54, His56, Lys86, Lys91). The widths of the DNA minor groove varies with sequence and can be a major determinant of DNA shape recognition by proteins^41^. For example, the NAP Fis protein selects targets primarily through indirect recognition mechanisms involving the shape of the minor groove (intrinsically narrow minor groove) and sequence-dependent induced fits over adjacent major groove interfaces. Fis-DNA X-ray structures have revealed that narrow minor grooves containing A/T-rich sequences are compressed to a width that is about half of that observed for canonical B-form DNA^42,43^ and that the neutralization of the proximal phosphates is due to the side chain of Lys90. In MC1, the minor groove compression is particularly important between C6 and T29 (just after the A-tract) and is facilitated by the neutralization of their phosphates by the side chain of Lys86 (Fig. 3b). Secondly, the kinked DNA^15bp^ V-turn conformation is stabilized by direct hydrophobic contacts via the intercalation or insertion of hydrophobic side chains (Pro72 and Trp74) into the minor groove. The resulted dramatic bend is made possible through the neutralization of the repulsive negative charges of the phosphates belonging to the narrow major groove by the Arg25 side chain with the help of Gln23 and Gln26 side chains (Fig. 3b). Thirdly, MC1 is able to stabilize a V-turn on a longer DNA because of a supplementary contact in the minor groove. The affinity of MC1 for this supplementary area is probably weak (non-specific interactions) resulting in an equilibrium between two different V-turn conformations of the MC1-DNA^23bp^ complex.

**Figure 7:**
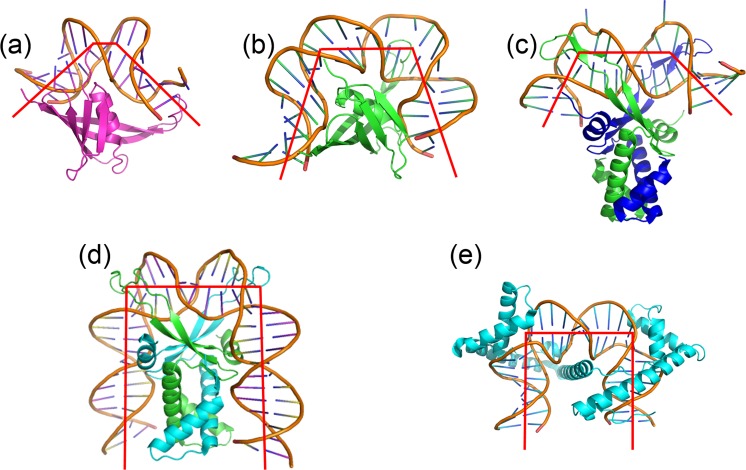
Cartoon representation of (**a**) MC1-DNA^15bp^ (PDB code 2NBJ), (**b**) model of MC1-DNA^23bp^, (**c**) HU-DNA (PDB code 1P71), (**d**) IHF-DNA (PDB code IHF) and, (**e**) TFAM-DNA (PDB code 3TMM) complexes. Red lines show the curvature of the DNA axis.

A few other DNA binding proteins are able to impose such a drastic DNA conformational change: two eukaryotic mitochondrial proteins (human TFAM^39,44^ and its yeast counterpart Abf2p^45^) and the prokaryotic nucleoid IHF^46,47^ and HU^48^ family proteins (Table 2, Fig. 7). At the atomic level, MC1, IHF, HU and TFAM use similar mechanisms to strongly bend DNA: binding in the minor groove, neutralization of the negative charges of the DNA backbone and intercalation of hydrophobic residues. However, the HMG boxes of TFAM interact into the convex face of the DNA curvature, whereas IHF/HU/MC1 interact with the concave side. IHF and TFAM create two almost coplanar kinks on DNA upon binding, leading to the DNA being bent by 163° and 180°, respectively, thus reversing the direction of the DNA helix forming a U-turn (Table 2). On the contrary, HU and MC1 induce two non-coplanar kinks, resulting in a medium (40–73°) or strong (106°) dihedral angle on the DNA. Torsion has a major impact on the overall bend angle. In the case of MC1 and HU, the bend angle values are then consistent with a V-turn DNA conformation.

**Table 2:** Comparison of the kinks angles, overall bend angles and dihedral angles for DNAs in complex with the MC1 (this work), HU^48^, IHF^46,47^ and TFAM^39,44^ proteins.

Protein-DNA complex	Kink 1 (°)	Kink 2 (°)	Overall bend (°)	Dihedral angle (°)	PDB code [ref]
MC1–15bp	55	70	66	106	2NBJ
AHU2–19bp	77	82	105	73	1P78^48^
TR3–19bp	74	84	106	72	1P71^48^
AHU6a-17bp	91	85	130	50	1P51^48^
AHU6b-17bp	89	82	139	40	1P51^48^
IHF-35bp	93	87	163	15	1IHF^46^
Hbb-35bp	~90	~90	~180	<5	2NP2^47^
TFAM-22bp	~90	~90	~180	0	3TQ6^44^
TFAM-28bp	~90	~90	~180	0	3TMM^39^

Our 3D structure helps us to understand how a small monomeric protein, such as MC1 with a completely asymmetric conformation, can sharply bend the DNA and impose a strong torsional angle. As previously known, MC1 is a bender but might also have a secondary binding mode as a wrapper protein. Indeed, some NAPs, as HU (bender and wrapper) or as Fis (bender and bridger) exhibit dual architectural properties that are likely dependent on protein concentration or DNA binding sequence^1^. In the IHF/HU complexes, under-twisting of the DNA near the kinks is partially compensated by over-twisting of the DNA between the kinks, whereas, in the MC1-DNA^15bp^, there is an overtwisting at the first kink and near the second kink, which is compensated by undertwisting between the kinks and after the second kink. This new mode of torsion is a key point to allow a small monomeric protein to impose a V-turn conformation on the DNA. The flexible and dynamic nature of the V-turn conformation can be modulated through the intrinsic dynamics of the protein (adaptability of its core through small local rearrangement and of its arm through a more or less extended conformation) and/or additional interactions with DNAs, such as those observed with the MC1-DNA^23bp^ complex. Indeed, MC1, contrary to IHF and like HU, binds DNA without sequence specificity and selects structural features of DNA for binding^17,49^.

More than just a histone-like protein involved in the maintenance of negative supercoiling and chromosomal compaction, the flexibility and dynamics of the MC1-DNA complexes observed in solution could also reveal anchor points (i.e. molecular targets) for other molecular partners involved in various DNA transactions. The formation of such higher-order protein-DNA architectures requires the conformation of the DNA template to be bent or distorted. Bending brings distant sites of the DNA into proximity, which is necessary for the site-directed recombination process, whereas negative supercoiling favors unwinding and is likely to facilitate processes in which proteins need access to the DNA bases, such as repair, replication and transcription molecular machineries.

The binding mode of MC1 results in the formation of a particularly widened minor groove in the A_7_pC_8_pA_9_/T_22_pG_23_pT_24_ region, permitting ready access to the DNA by other DNA-binding proteins. For example, architectural DNA-binding proteins can modulate the DNA glycosylase activity. DNA glycosylases, which initiate the base excision repair (BER) process, recognize and extrude the damaged base, stabilizing it into an extrahelical conformation inside the active site pocket. To achieve this base-flipping mechanism, DNA at the lesion site is bent by inserting an intercalating residue triad inside the minor groove. The damaged nucleotide is then expelled from the DNA double helix by the major groove. We previously showed that the *E. coli* HU could stimulate the turnover of the Formamidopyrimidin-DNA glycosylase (Fpg)^50^. Like HU, MC1 could play a role in DNA repair *in vivo* in Archaea.

The knowledge of which proteins are involved in modulating chromatin structure in archaeal organisms is incomplete, and views on the interplay between chromatin proteins, such as MC1, and transcription are still unknown. The archaeal transcription machinery is closely related to the RNA polymerase II system in terms of subunit composition, structure, use of general factors and molecular mechanisms^8^. There are two putative transcriptional regulatory mechanisms involving chromatin proteins in Archaea. The first involves histone proteins, which compete for DNA-binding to the promoter. Their steric and torsional effects limit the binding of basal transcription factors to the DNA^8,51^. *In vitro*, the *E. coli* RNA polymerase activity is stimulated by the addition of MC1 at low protein to DNA ratios, but is inhibited at higher ratios^22^. Such a bell-shape effect was also observed in the experiments showing the stimulation of Fpg by HU^50^. We propose that MC1 uses the same transcriptional regulatory mechanism of competition as histones. The second mechanism involves the post-translational modification of NAPs, especially acetylation of the Alba protein^6^, which modifies their DNA-binding properties. MC1-α from *Methanosarcina mazei* strain Gö1 was described to be specifically methylated (Lys37) by the methyltransferase Gö1-SET, *in vitro*^52^. In MC1, this Lys37 is replaced by a serine, located into the loop between the helix and the β3-strand, a region which has not been implicated in the interaction surface of the MC1-DNA^15bp^ complex. Among the MC1 family proteins, this loop presents a great variability in the number and nature of amino acids (see alignment in ref.^29^). It often contains at least one lysine, which could be methylated. Since methylation does not alter the DNA-binding interface of MC1-α, the role of this modification needs to be clarified. Cren7 and Sul7 are also both known to be methylated at several lysine residues without any known impact *in vitro*^53^. Such post-translational modifications of MC1-α, Sul7, Cren7, and thus MC1, can alter their affinity for other molecular partners.

Finally, the V-shape structure of the MC1-DNA complexes provides a new perspective for the high affinity of MC1 to four-way junctions^27^. Indeed, cruciform structures are fundamentally important for a wide range of biological processes, including replication and recombination. MC1 has been shown to be involved in cellular division in promoting replication and high rates of growth in the psychrophilic methanogen *Methanococcoides burtonii* after heat-shock at 23 °C^23,24^. In *Halobacterium salinarum*, a growth-phase-dependent effect on the relative expression of MC1 and histone proteins was shown^54^. The Holliday junction-resolvase Hjc, conserved in archaea, specifically recognizes four-way DNA junctions, cleaving them without sequence preference to generate recombinant DNA duplex. Hjc, which is retro-inhibited by its Hollyday-junction cleavage product (no turnover *in vitro*) is released by the addition of the architectural double-stranded DNA-binding protein Sso7d^55^. MC1 could regulate the Hjc enzymatic activity in methanosarcinal, as Sso7d does in *Sulfolobus*^55^.

In summary, we have determined the 3D NMR structure of a MC1-DNA^15bp^ complex showing the remarkable ability of a small and monomeric protein to induce DNA bending and DNA torsional constraints. Our structural work highlights a new and very efficient way, used by MC1, to produce a DNA V-turn. Indubitably, MC1 largely contributes to the organization of the genome of methanosarcinal and haloarchaeal classes of *Euryarchaea*. The structural determination of MC1-DNA complexes opens up new opportunities for studying and understanding the different emerging roles of MC1 to modulate DNA accessibility to transcription and replication machineries.

## Materials and Methods

### NMR sample preparation

The MC1-DNA^15bp^ complex samples (20 kDa) were prepared in 10 mM phosphate buffer pH 6, 100 mM NaCl, 1 mM EDTA, 10% D_2_O and were previously described: ^15^N MC1-DNA^15bp^ complex^29^ (1 mM) and ^15^N MC1-^13^C^15^N DNA^15bp^ complex (1.18 mM)^56^. The first strand (5′- A_1_AAAACACACACCCA_15_ −3′) is uniformly ^15^N and ^13^C-labelled and the second strand (5′- T_16_GGGTGTGTGTTTTT_30_ −3′) is in natural abundance. To measure residual dipolar couplings, the filamentous bacteriophage Pf1 (50 mg/ml, Asla Biotech. Ltd., Latvia) was added in the ^15^N MC1-^13^C^15^N DNA^15bp^ complex sample as the orienting medium. The corresponding final concentration was ^15^N MC1-^13^C^15^N DNA^15bp^ complex (0.85 mM) with Pf1 bacteriophage (10 mg/ml).

The ^15^N MC1-DNA^23bp^ complex sample (25 kDa) was prepared in the same way to obtain a final concentration of 0.67 mM.

### NMR experiments and structure calculations of the MC1-DNA^15bp^ complex

All NMR experiments were performed at 298 K on a 700 MHz Bruker Avance III HD spectrometer equipped with a cryoprobe. All data were processed using Topspin Bruker and assignments were performed with the CcpNmr suite^57^. Interproton distances were derived from NOESY data sets run at 120 ms mixing time. ^1^D_NH_ RDCs were measured by using 2D IPAP ^1^H-^15^N and ^1^H-^13^C HSQC experiments acquired on a 600 MHz Varian ^UNITY^INOVA spectrometer for both the isotropic and anisotropic conditions^32^.

Structures were calculated with NOE distance, hydrogen-bond, and RDC constraints using the HADDOCK web server based on restrained MD/SA (XPLOR/CNS)^58^. The H-bond restraints used for the calculation of the 3D structures (Table 1) were deduced from the cross peaks observed in the NOESY spectrum. For MC1, they were set in accordance with the observation of typical long or medium NOE cross peaks network for β-sheets and α-helices respectively – H^N^/H^N^, H^N^/H^α^, H^α^/H^α^. For DNA, they were set in accordance with the observation of NOE cross peaks between the H1 imino protons of the guanines and the H4 amino protons of the cytosines and, between the H3 imino protons of the thymines and the H2 aromatic protons of the adenines. Our precedent model, obtained by data-driven docking^29^, allowed us to calculate the expected protein-DNA contacts which helped us to unambiguously identify 50 intermolecular distance restraints derived from NOEs. RDC restraints were incorporated at the last iteration with the proper parameters for MC1 (Da = 18.94 and R = 0.58) and DNA (Da = −13.8 and R = 0.36). RDC restraints were fitted and analyzed with the MODULE program^59^.

Docking was started from the whole ensemble of the 15 lowest-energy MC1 free structures (2KHL.pdb) and a B-standard DNA structure obtained with 3D-DART^60^. The first step of the protocol^58^ described for a protein-DNA complex which consisted of a rigid-body docking (1000 models) followed by a refinement stage (200 models). Both protein and DNA were successively defined to be fully flexible on all their length (first run), then fully flexible for residues 63–80 (MC1) and 12–15/16–18 (DNA^15bp^) and finally automatically semi-flexible throughout the different runs, 40 in total. The final step of the structure refinement was performed in explicit water (200 models). The twelve structures with the lowest interaction energies and lowest constraints violations were selected for further analysis. Protein analysis was performed with Procheck. The figures were prepared with PyMOL^61^ and Molmol^62^.

### Relaxation experiments

^1^H-^15^N CPMG experiments were recorded with ν_CPMG_ = 0, 50, 100, 150, 200, 250, 300, 350, 400, 500, 600, 700, 800 and 900 Hz, and a CPMG constant time delay, T_cp_ of 80 ms and 40 ms for MC1 and MC1-DNA^23bp^, respectively^63^. Peak intensities were plotted as effective relaxation rates and relaxation dispersion curves were fitted using the NESSY software^64^.

### Model construction of the MC1-DNA^23bp^ complex

DNA^23bp^ was constructed with 3D-Dart starting from bound DNA^15bp^ structure and a canonical B-DNA of 8 bp was added to the terminal A_15_T_16_ base pair. By substituting DNA^23bp^ in the MC1-DNA^15bp^ complex structure and minimization, we obtained a model of the MC1-DNA^23bp^ complex.

### Electrophoretic mobility-shift assays

Two ^32^P-labeled DNA duplexes *DNA^23bp^ and *DNA^15bp^, containing the NMR used sequences with an additional base pair at each extremity, were respectively prepared by annealing a 25-mer (5′-CAAAAACACACACCCAAACTAACAG) and a 17-mer (5′-CAAAAACACACACCCAG) oligonucleotide to their complementary strand, as previously described^20^. EMSA reaction mixtures (10 µl) were prepared at 4 °C by mixing DNA duplexes (0.05 nM) and increasing MC1 protein concentrations from 0.04 nM to 6.6 nM, in binding buffer (10 mM Tris–HCl, 400 mM NaCl, 200 µg.ml^−1^ BSA, and 10% (v/v) glycerol, pH 7.5), followed by incubation for 30 min at 4 °C. The different mixtures were then loaded onto a 12% polyacrylamide gel (29:1 acrylamide:bisacrylamide) in 0.5 TBE buffer (44.5 mM Tris-HCl, pH 8.3, 44.5 mM boric acid, 0.5 mM EDTA). Electrophoresis was run at 14 V/cm, for 2 hours at 10 °C. After drying, gels were scanned with a β-scanner (Typhoon Trio) and quantified using ImagQuant software. The binding curves were fitted to a single binding site model using the equation Y = [MC1]/([MC1] + K_Dapp_) where Y is the fraction of bound DNA, [MC1] is the concentration of free protein, and K_Dapp_ is the apparent dissociation constant. Under our experimental conditions, the free protein concentration is reasonably approximated by the total protein concentration. The obtained values correspond to the mean (±SD) of three independent experiments.

### Assession numbers

NMR restraints and atomic coordinates for the MC1-DNA^15bp^ complex have been deposited with the RCSB PDB under the accession code PDB ID: 2NBJ.

## References

[1] Luijsterburg MS, White MF, van Driel R, Dame RT (2008). The major architects of chromatin: architectural proteins in bacteria, archaea and eukaryotes. Crit. Rev. Biochem. Mol. Biol..

[2] Sandman K, Krzycki JA, Dobrinski B, Lurz R, Reeve JN (1990). HMf, a DNA-binding protein isolated from the hyperthermophilic archaeon Methanothermus fervidus, is most closely related to histones. Proc. Natl. Acad. Sci. USA.

[3] Starich MR, Sandman K, Reeve JN, Summers MF (1996). NMR structure of HMfB from the hyperthermophile, Methanothermus fervidus, confirms that this archaeal protein is a histone. Journal of molecular biology.

[4] Sandman K, Reeve JN (2005). Archaeal chromatin proteins: different structures but common function?. Curr. Opin. Microbiol..

[5] Laurens N (2012). Alba shapes the archaeal genome using a delicate balance of bridging and stiffening the DNA. Nat. Commun..

[6] Goyal M, Banerjee C, Nag S, Bandyopadhyay U (2016). The Alba protein family: structure and function. Biochim. Biophys. Acta.

[7] Marc F, Sandman K, Lurz R, Reeve JN (2002). Archaeal histone tetramerization determines DNA affinity and the direction of DNA supercoiling. J. Biol. Chem..

[8] Peeters E, Driessen RP, Werner F, Dame RT (2015). The interplay between nucleoid organization and transcription in archaeal genomes. Nat. Rev. Microbiol..

[9] White MF, Bell SD (2002). Holding it together: chromatin in the Archaea. Trends Genet..

[10] Robinson H (1998). The hyperthermophile chromosomal protein Sac7d sharply kinks DNA. Nature.

[11] Chen CY (2005). Probing the DNA kink structure induced by the hyperthermophilic chromosomal protein Sac7d. Nucleic Acids Res..

[12] Guo L (2008). Biochemical and structural characterization of Cren7, a novel chromatin protein conserved among Crenarchaea. Nucleic Acids Res..

[13] DeLange RJ, Williams LC, Searcy DG (1981). A histone-like protein (HTa) from Thermoplasma acidophilum. II. Complete amino acid sequence. J. Biol. Chem..

[14] Orfaniotou F (2009). The stability of the archaeal HU histone-like DNA-binding protein from Thermoplasma volcanium. Extremophiles: life under extreme conditions.

[15] Pontiggia A, Negri A, Beltrame M, Bianchi ME (1993). Protein HU binds specifically to kinked DNA. Mol. Microbiol..

[16] Castaing B, Zelwer C, Laval J, Boiteux S (1995). HU protein of Escherichia coli binds specifically to DNA that contains single-strand breaks or gaps. J. Biol. Chem..

[17] Kamashev D, Balandina A, Rouviere-Yaniv J (1999). The binding motif recognized by HU on both nicked and cruciform DNA. EMBO J..

[18] Chartier, F., Laine, B. & Sautiere, P. Characterization of the chromosomal protein MC1 from the thermophilic archaebacterium Methanosarcina sp. CHTI 55 and its effect on the thermal stability of DNA. *Biochim. Biophys. Acta* **951**, 149–156; 0167-4781(88)90035-8 (1988). 10.1016/0167-4781(88)90035-8 3142520

[19] Le Cam E, Culard F, Larquet E, Delain E, Cognet JA (1999). DNA bending induced by the archaebacterial histone-like protein MC1. Journal of molecular biology.

[20] Teyssier C (1996). Preferential binding of the archaebacterial histone-like MC1 protein to negatively supercoiled DNA minicircles. Biochemistry.

[21] Toulme F (1995). Conformational changes of DNA minicircles upon the binding of the archaebacterial histone-like protein MC1. J. Biol. Chem..

[22] Chartier F, Laine B, Belaiche D, Touzel JP, Sautiere P (1989). Primary structure of the chromosomal protein MC1 from the archaebacterium Methanosarcina sp. CHTI 55. Biochim. Biophys. Acta.

[23] Williams TJ (2010). Global proteomic analysis of the insoluble, soluble, and supernatant fractions of the psychrophilic archaeon Methanococcoides burtonii. Part I: the effect of growth temperature. J. Proteome Res..

[24] Goodchild A, Raftery M, Saunders NF, Guilhaus M, Cavicchioli R (2004). Biology of the cold adapted archaeon, Methanococcoides burtonii determined by proteomics using liquid chromatography-tandem mass spectrometry. J. Proteome Res..

[25] Paquet F, Culard F, Barbault F, Maurizot JC, Lancelot G (2004). NMR solution structure of the archaebacterial chromosomal protein MC1 reveals a new protein fold. Biochemistry.

[26] Paquet F (2010). Refined solution structure and backbone dynamics of the archaeal MC1 protein. FEBS J..

[27] Paradinas C, Gervais A, Maurizot JC, Culard F (1998). Structure-specific binding recognition of a methanogen chromosomal protein. Eur. J. Biochem..

[28] Isabelle V (1993). Radioprotection of DNA by a DNA-binding protein: MC1 chromosomal protein from the archaebacterium Methanosarcina sp. CHTI55. Int. J. Radiat. Biol..

[29] Paquet F (2014). Model of a DNA-protein complex of the architectural monomeric protein MC1 from Euryarchaea. PloS one.

[30] De Vuyst G, Aci S, Genest D, Culard F (2005). Atypical recognition of particular DNA sequences by the archaeal chromosomal MC1 protein. Biochemistry.

[31] Vermeulen A, Zhou H, Pardi A (2000). Determining DNA global structure and DNA bending by application of NMR residual dipolar couplings. J. Am. Chem. Soc..

[32] Hansen MR, Hanson P, Pardi A (2000). Filamentous bacteriophage for aligning RNA, DNA, and proteins for measurement of nuclear magnetic resonance dipolar coupling interactions. Methods Enzymol..

[33] Rohs R (2010). Origins of specificity in protein-DNA recognition. Annu. Rev. Biochem..

[34] Blanchet C, Pasi M, Zakrzewska K, Lavery R (2011). CURVES+ web server for analyzing and visualizing the helical, backbone and groove parameters of nucleic acid structures. Nucleic Acids Res..

[35] Lavery R, Moakher M, Maddocks JH, Petkeviciute D, Zakrzewska K (2009). Conformational analysis of nucleic acids revisited: Curves+. Nucleic Acids Res..

[36] Lu XJ, Olson WK (2003). 3DNA: a software package for the analysis, rebuilding and visualization of three-dimensional nucleic acid structures. Nucleic Acids Res..

[37] Laine B, Culard F, Maurizot JC, Sautiere P (1991). The chromosomal protein MC1 from the archaebacterium Methanosarcina sp. CHTI 55 induces DNA bending and supercoiling. Nucleic Acids Res..

[38] Saenger, W. *Principles of nucleic acid structure*. (Springer-Verlag, 1984).

[39] Ngo HB, Kaiser JT, Chan DC (2011). The mitochondrial transcription and packaging factor Tfam imposes a U-turn on mitochondrial DNA. Nat. Struct. Mol. Biol..

[40] Kleckner IR, Foster MP (2011). An introduction to NMR-based approaches for measuring protein dynamics. Biochim. Biophys. Acta.

[41] Rohs R (2009). The role of DNA shape in protein-DNA recognition. Nature.

[42] Hancock SP (2013). Control of DNA minor groove width and Fis protein binding by the purine 2-amino group. Nucleic Acids Res..

[43] Stella S, Cascio D, Johnson RC (2010). The shape of the DNA minor groove directs binding by the DNA-bending protein Fis. Genes Dev..

[44] Rubio-Cosials A (2011). Human mitochondrial transcription factor A induces a U-turn structure in the light strand promoter. Nat. Struct. Mol. Biol..

[45] Chakraborty A (2017). DNA structure directs positioning of the mitochondrial genome packaging protein Abf2p. Nucleic Acids Res..

[46] Rice PA, Yang S, Mizuuchi K, Nash HA (1996). Crystal structure of an IHF-DNA complex: a protein-induced DNA U-turn. Cell.

[47] Mouw KW, Rice PA (2007). Shaping the Borrelia burgdorferi genome: crystal structure and binding properties of the DNA-bending protein Hbb. Mol. Microbiol..

[48] Swinger KK, Lemberg KM, Zhang Y, Rice PA (2003). Flexible DNA bending in HU-DNA cocrystal structures. EMBO J..

[49] Kamashev D, Rouviere-Yaniv J (2000). The histone-like protein HU binds specifically to DNA recombination and repair intermediates. EMBO J..

[50] Le Meur R (2015). The nucleoid-associated protein HU enhances 8-oxoguanine base excision by the formamidopyrimidine-DNA glycosylase. Biochem. J..

[51] Gehring AM, Walker JE, Santangelo TJ (2016). Transcription regulation in Archaea. J. Bacteriol..

[52] Manzur KL, Zhou MM (2005). An archaeal SET domain protein exhibits distinct lysine methyltransferase activity towards DNA-associated protein MC1-alpha. FEBS Lett..

[53] Driessen RP, Dame RT (2013). Structure and dynamics of the crenarchaeal nucleoid. Biochem. Soc. Trans..

[54] Dulmage KA, Todor H, Schmid AK (2015). Growth-phase-specific modulation of cell morphology and gene expression by an archaeal histone protein. mBio.

[55] Kvaratskhelia M (2002). Holliday junction resolution is modulated by archaeal chromatin components *in vitro*. J. Biol. Chem..

[56] Loth K, Landon C, Paquet F (2015). Chemical shifts assignments of the archaeal MC1 protein and a strongly bent 15 base pairs DNA duplex in complex. Biomol. NMR Assign..

[57] Vranken WF (2005). The CCPN data model for NMR spectroscopy: development of a software pipeline. Proteins.

[58] de Vries SJ, van Dijk M, Bonvin AM (2010). The HADDOCK web server for data-driven biomolecular docking. Nat. Protoc..

[59] Dosset P, Hus JC, Marion D, Blackledge M (2001). A novel interactive tool for rigid-body modeling of multi-domain macromolecules using residual dipolar couplings. J. Biomol. NMR.

[60] van Dijk M, Bonvin AM (2009). 3D-DART: a DNA structure modelling server. Nucleic Acids Res..

[61] De Lano, W. L. *Pymol*. (De Lano Scientific, 2002).

[62] Koradi, R., Billeter, M. & Wuthrich, K. MOLMOL: a program for display and analysis of macromolecular structures. *J. Mol. Graphics* **14**, 51–55, 29-32;0263785596000094 [pii] (1996). 10.1016/0263-7855(96)00009-4 8744573

[63] Tollinger M, Skrynnikov NR, Mulder FA, Forman-Kay JD, Kay LE (2001). Slow dynamics in folded and unfolded states of an SH3 domain. J. Am. Chem. Soc..

[64] Bieri M, Gooley PR (2011). Automated NMR relaxation dispersion data analysis using NESSY. BMC Bioinformatics.

